# Regulating digital surgery to balance safety and innovation: a SAGES white paper

**DOI:** 10.1007/s00464-026-12875-6

**Published:** 2026-06-19

**Authors:** Natalie M. Guzman, Jayson S. Marwaha, Deborah S. Keller, Liane S. Feldman, Daniel A. Hashimoto, Amin Madani, Dana A. Telem

**Affiliations:** 1 Department of Learning Health Sciences, University of Michigan, Ann Arbor, MI USA; 2 Center for Healthcare Outcomes and Policy, University of Michigan, Ann Arbor, MI USA; 3 Department of Surgery, University of Michigan, Ann Arbor, MI USA; 4 Arizona State University, Tempe, AZ USA; 5 Mayo Clinic, Phoenix, AZ USA; 6 Department of Surgery, McGill University, Montreal, QC Canada; 7 Department of Surgery, Hospital of the University of Pennsylvania, Philadelphia, PA USA; 8 Department of Surgery, University of Toronto, Toronto, ON Canada; 9 Department of Surgery, Section of General Surgery, University of Michigan Health Systems, 1500 E. Medical Center Drive, Ann Arbor, MI 48109-0340 USA

**Keywords:** Digital surgery, Artificial intelligence, Regulatory framework, Medical device regulation, Patient safety

## Abstract

**Background:**

Digital surgery technologies, including robotic systems, artificial intelligence (AI) algorithms, augmented reality platforms, and advanced data analytics, are rapidly transforming surgical practice. While these technologies hold tremendous potential to improve patient outcomes, they introduce unique regulatory challenges that current frameworks are not fully equipped to address.

**Methods:**

This white paper from the SAGES Surgical Data Science Committee characterizes the regulatory challenges specific to digital surgery technologies and proposes general regulatory principles designed to balance patient safety with efficient delivery of beneficial innovations.

**Results:**

We identify key regulatory gaps across three categories of digital surgery technologies, including enhanced instrumentation and robotics, advanced visualization systems, and AI data analytics and capture. Unlike diagnostic tools, these technologies guide real-time, irreversible actions where errors can cause immediate and permanent patient harm. Critical challenges include the lack of clinically meaningful performance metrics, ambiguous liability across hardware and software stakeholders, performance drift in adaptive algorithms, algorithmic bias, data governance concerns, and insufficient cybersecurity standards for intraoperative systems. To address these gaps, we propose a three-tier risk-stratified classification framework aligned with existing FDA pathways, guidance on predetermined change control plans for adaptive technologies, recommendations for performance metrics that balance technical and clinical evaluation, established evaluation frameworks such as IDEAL and stage-specific reporting guidelines to structure development and assessment, a defined role for professional societies and collaborative communities in shaping clinically relevant standards, and evidence requirements reflecting the procedural context of these tools.

**Conclusions:**

Regulation of digital surgery must balance innovation with safety, recognizing that both excessive and insufficient oversight can harm patients. Traditional metrics and trial designs are insufficient, as evaluation must be anchored in clinically meaningful outcomes, ongoing surveillance, and real-world validation. The framework presented provides a foundation for achieving this balance through risk-proportionate, evidence-based regulatory approaches.

Digital surgery represents a paradigm shift in surgical practice that inserts a computer interface between the surgeon and patient, reshaping how surgical procedures are performed, monitored, and assessed [1]. Robotic surgical systems can enable minimally invasive procedures with enhanced dexterity and control, while artificial intelligence (AI) algorithms will be able to provide real-time analysis of surgical video feeds to identify critical anatomy and potential complications [2, 3]. Similarly, augmented reality (AR) systems can overlay digital information onto the surgical field, helping to guide instrument placement and improve navigation accuracy [4–6]. Additionally, machine learning (ML) models can enable the analysis of large datasets of surgical outcomes to predict patient risk and optimize treatment plans [7–9].

While these technologies have tremendous potential to transform surgical care, they also introduce significant risks. AI algorithms may provide incorrect recommendations during critical procedures, AR systems may display inaccurate visualizations that mislead surgeons, and robotic systems can malfunction during operations. Machine learning models may exhibit bias against certain patient populations, and autonomous surgical systems could make life-threatening errors without human oversight [10, 11].

AI, when incorporated into surgical and procedural technologies, carries distinct risks because unlike diagnostic tools, they guide real-time, irreversible actions, where there is no time to cross-check and correct incorrect recommendations. Additionally, like other AI-based technologies, they often learn and adapt over time, integrate hardware and software components, process real-time data streams, and require the characterization of metrics for measuring performance and safety [12]. As these systems become more sophisticated, they will require equally sophisticated regulatory approaches capable of ensuring thorough evaluation of high-risk technologies while avoiding unnecessarily burdensome requirements for low risk tools.

In this paper, we characterize the unique regulatory challenges of digital surgery technologies. Following this discussion, we provide guidance on general regulatory principles specific to digital surgery technologies that enable safe implementation while allowing for efficient, expedited delivery of these beneficial technologies to patients.

## Digital surgery regulatory challenges

### Enhanced instrumentation and robotics

Enhanced surgical instruments and robotics represent a fundamental transformation in medical technology. These technologies include powered staplers with tissue compression sensing, advanced energy devices with impedance-based feedback, and roboticized devices that integrate robotic technology into traditional surgical instruments [1]. Until recently, surgical tools have been static instruments that simply extend the surgeon's capabilities, but we now have access to intelligent systems equipped with sensors, automation capabilities, and varying levels of autonomy [1, 13]. This evolution has created unprecedented regulatory challenges, as current device classification systems were designed for medical devices with fixed functionality rather than hybrid hardware-software systems that integrate artificial intelligence technology [14]. The United States Food and Drug Administration (FDA) is moving toward addressing these challenges and recently issued final guidance providing recommendations for predetermined change control plans (PCCPs) tailored to AI-enabled devices [15]. Yet, significant challenges persist.

The lack of clinically relevant and validated performance metrics represents a critical regulatory gap for enhanced instrumentation. While various performance metrics exist for these adaptive technologies, current regulatory frameworks often rely on technical benchmarks that may not translate to meaningful clinical outcomes or patient safety measures, making it difficult to assess real-world safety and efficacy consistently across different devices and clinical applications. This challenge is compounded by evidence that validation metrics in AI healthcare technology are often inadequately chosen and poorly correlated with clinical outcomes, which can hinder the translation of these tools into clinical practice [16]. Without metrics grounded in outcome data, regulators cannot effectively measure device performance, compare competing technologies, or establish meaningful safety thresholds for approval [17]. Performance metrics must account for both the mechanical components and the software algorithms that control the device’s actions, requiring evaluation methodologies that can assess integrated systems in a sophisticated manner.

While the FDA’s PCCP guidance for AI-enabled device software functions represents an important step forward in addressing software modifications and the continuous learning nature of these systems, there is still ambiguity to be addressed. When there is collaboration between device manufacturers and software developers in creating these hybrid systems, this leads to ambiguous liability scenarios that current regulations fail to address, leaving unclear responsibilities for device performance, safety monitoring, and adverse event reporting across the various stakeholders involved in these integrated technologies [18, 19].

### Advanced visualization technologies

Advanced visualization systems build upon the challenges of enhanced instrumentation while introducing unique complexities around real-time image processing, registration accuracy, human factors considerations, and cybersecurity [1, 20–24]. These technologies include three-dimensional visualization, fluorescence-guided surgery, and AR systems that provide real-time visual information overlaid on the surgical field [1]. Similarly to enhanced instrumentations and robotics, the adaptive nature of these technologies challenges traditional performance metrics [25]. Visualization systems rely on real-time image processing algorithms that must continuously adapt to changing surgical conditions, varying patient anatomy, and different lighting environments. This creates uncertainty about establishing appropriate safety thresholds for technologies whose output varies based on contextual factors that cannot be fully predicted during pre-market testing and can only be fully appreciated upon implementation. [26].

Registration accuracy represents one of the most critical performance metrics, particularly for AR applications where virtual objects must align precisely with real anatomy [20]. Registration failures can lead directly to surgical errors with potentially harmful outcomes [27]. Current regulatory frameworks lack standardized metrics for evaluating registration performance, and this challenge is compounded by the absence of established quality metrics for registration evaluation itself. This problem becomes even more complex in fields like general surgery where the target anatomy is not rigid, but instead deforms in a dynamic manner (i.e., deformations with heartbeat and respiration) [1, 20].

These systems also present challenges for clinical trial methodologies. Unlike traditional devices evaluated with standardized protocols, visualization systems or any technologies or procedures where surgeon and patient factors affect the outcome must be assessed within their intended clinical environments where real-world factors, such as a surgeon’s skill and the real data source, significantly influence performance [28].

This necessitates evaluation methodologies extending beyond traditional randomized controlled trials to include comprehensive implementation studies and real-world evidence collection [29]. While this will be discussed in greater details in subsequent sections, the Idea, Development, Exploration, Assessment and Long-term monitoring (IDEAL) framework can also guide in determining the types of studies and reporting needed for new surgical procedures and technologies such as AR systems [30]. This framework recognizes that surgical innovations require staged evaluation approaches that account for the learning curves, technical variations, and contextual factors that influence real-world implementation and outcomes [30–33].

Human factors considerations present another related regulatory gap, as advanced visualization systems directly impact surgeon cognitive load, visual attention, and decision-making processes [21, 23, 24, 34]. Even minor side effects like fatigue, disorientation, or visual obstruction could become dangerous during critical surgical moments [35].

Additionally, cybersecurity concerns arise from the networked infrastructure required for image processing and data storage, resulting in potential exposures that could compromise patient safety [36]. Current regulatory frameworks could be improved with comprehensive cybersecurity standards specifically tailored to real-time surgical visualization systems, where security breaches could have immediate and severe consequences during active procedures.

## AI data analytics and data capture

AI systems integrated with comprehensive data analytics and capture represent one of the most complex regulatory challenges in digital surgery. This encompasses real-time surgical decision support algorithms, predictive analytics for patient outcomes, automated surgical video analysis, and extensive perioperative data collection platforms that capture everything from instrument kinematics to surgeon performance metrics [1]. In addition to some of the factors already discussed, AI systems pose multiple regulatory challenges.

A known reality of these models is performance drift. Unlike traditional devices where performance degradation typically results from mechanical wear or component failure, AI systems experience performance drift when real-world data differs from training data, when patient populations change, or when clinical workflows evolve [37–40]. Current regulatory frameworks lack adequate direction on mechanisms for detecting, monitoring, and responding to such performance drift.

Again, the issue of meaningful metrics arises. It is unclear if developers should be prioritizing algorithmic performance measures like sensitivity and specificity or clinical performance indicators like surgical outcomes and patient safety, or more realistically, a balance of both [16, 41]. Even within sensitivity and specificity measures, metrics can be manipulated to prioritize different analytical targets, such as voxel-level sensitivity for algorithmic performance versus lesion-level sensitivity for clinical relevance in the field of medical imaging [42, 43]. Additionally, different outcomes may need to be prioritized at different stages of development. The choice of evaluation metrics affects how these systems are assessed, approved, and monitored, yet a standardized approach for determining which metrics are appropriate for different AI applications in digital surgery does not exist. This ambiguity may lead to approval of systems that perform well on technical metrics but fail to improve clinical outcomes, compromising patient safety.

Algorithmic bias presents another important regulatory gap. AI systems can perpetuate or amplify existing healthcare disparities when training data lacks diversity or when algorithms are optimized for majority populations [44, 45]. In surgical applications, this could mean that decision support systems perform well for certain demographic groups while providing suboptimal recommendations for others, potentially exacerbating existing inequities in surgical care.

Data capture and ownership issues also challenge traditional regulatory frameworks. Digital surgery platforms can generate unprecedented amounts of data, including surgical video recordings, instrument kinematics, audio recordings, and comprehensive procedure documentation [1]. This data has immense value for quality improvement, surgeon training, and algorithm development, but there are still many questions related to data governance, ownership rights, privacy protection, and appropriate use limitations [46]. Current legal and regulatory frameworks like the Health Insurance Portability and Accountability Act (HIPAA) may not adequately address data governance considerations in the modern era, where we should prioritize data sharing approaches that preserve patient, clinician, and institutional privacy while enabling more comprehensive analysis of health phenomena that could potentially only be captured through larger, more diverse datasets.

Looking to the future, federated learning may offer a scalable, robust, and secure solution for real-world medical diagnostics, enabling healthcare institutions to train highly accurate models without compromising sensitive patient data [47]. With federated learning, multiple medical centers can leverage each other's learning without ever sharing sensitive patient data, ensuring privacy while enhancing collaborative learning [47]. This approach could address some current data governance challenges while supporting the development of more effective digital surgery technologies.

## General principles for regulating digital surgery

### Existing regulatory landscape of digital surgery

Digital surgery and procedural technologies have unique risk considerations that differ from other digital tools in medicine. Procedural technologies provide guidance for irreversible actions, unlike diagnostic decision support systems where it is more feasible that incorrect recommendations could be identified and corrected. For example, if a surgeon had technology that incorrectly guides them to cut the bile duct or misidentifies critical anatomy during a cardiac procedure, the consequences are immediate and, in many cases, life-threatening. This procedural versus non-procedural distinction require regulatory approaches more aligned with current medical device requirements than traditional software evaluation, which is the path we are seeing.

The FDA currently regulates digital surgery technologies through 510(k) clearance for substantially equivalent devices, De Novo classification for novel low-to-moderate risk devices, and pre-market approval (PMA) for high-risk devices [37, 48]. Current examples demonstrate how different types of digital surgery technologies have navigated these pathways successfully. For instance, navigation systems like Proprio's Paradigm AI Surgical Guidance Platform and robotic systems like Moon Surgical's Maestro System with AI-powered ScoPilot have received 510(k) clearance, demonstrating substantial equivalence to existing technologies [49, 50]. Meanwhile, novel robotic systems without clear predicates, such as Virtual Incision’s MIRA Surgical System for miniaturized robotic-assisted surgery and CMR Surgical’s Versius Surgical Robotic System, have successfully navigated the De Novo pathway [51, 52]. The Pre-market Approval (PMA) pathway remains reserved for the highest-risk innovations, as exemplified by Perimeter Medical Imaging's B-Series OCT with ImgAssist AI 2.0, which is currently under FDA review for real-time surgical margin assessment during breast-conserving surgeries [53]. Currently, PMA is rare for AI surgical tools.

Given the specific regulatory challenges with digital surgery tools and technologies, it is important to consider how these systems require different regulatory approaches than traditional medical devices. While these FDA pathways provide a foundation, they inadequately address some of the unique complexities specific to digital surgery and procedural technologies. The America’s AI Action Plan, the FDA's AI/ML Software as a Medical Device Action Plan, and PCCPs represent important steps forward, but we will have to further tailor our regulatory guidance to ensure efficient and safe regulations of these tools [14, 48, 54, 55]. Building on the FDA's existing frameworks, we propose a regulatory structure that incorporates enhanced risk-based principles specifically designed for the procedural context of digital surgery and to account for the fact that errors in these applications can carry immediate and irreversible patient harm (Table 1).

**Table 1:** Digital surgery regulatory framework: risk-stratified approach

Risk category	Technology examples	Regulatory pathway	Evidence requirements	Ongoing monitoring
Low risk: Minimal potential for direct patient harm. Technologies that assist in planning, analysis, or workflow without direct procedural guidance	• Aidoc briefcase: AI reads CT scans for incidental findings relevant to surgical • Viz vascular suite: AI detection of vascular pathologies for workflow prioritization • Avicenna. AI CINA chest: Automated detection of pulmonary embolism and aortic dissection on CT angiography	Streamlined 510(k) • Predicated on legally marketed device • Basic safety validation • User interface validation • Expedited review timelines	Pre-market: • Usability testing (establish minimal acceptable performance thresholds) • Basic cybersecurity assessment Clinical evidence: • User acceptance studies	Reporting: • Adverse event reporting • User feedback collection Performance: • Basic performance metrics • Annual performance summaries
Moderate risk: Systems providing real-time guidance requiring human oversight. Technologies that provide procedural assistance but maintain surgeon control	• Proprio paradigm: AI-guided visualization and real-time intraoperative measurements for spine surgery • Moon surgical maestro system: AI-powered ScoPilot for laparoscope control during minimally invasive procedures • Navigation systems with real-time guidance capabilities	De Novo or 510(k) • Comprehensive pre-market evaluation • Clinical validation studies • Human factors assessment • Algorithmic bias evaluation • Workflow integration studies • Learning curve	Pre-market: • Clinical studies (establish minimal number of procedures done) • Multi-site validation • Human factors validation • Performance across diverse population • Cybersecurity assessment Clinical Evidence: • Non-inferiority to standard of care • Workflow integration validation	Reporting: • Adverse event reporting • Quarterly performance reports • Real-world data collection • Algorithm performance monitoring Performance: • Predefined intervention thresholds to recall from market • Bias detection monitoring • User competency tracking
High risk: Autonomous/semi-autonomous systems or technologies that can independently initiate irreversible actions. Novel devices without clear predicates	• MIRA surgical system: First miniaturized robotic-assisted surgery device for colectomy (De Novo) • Versius surgical robotic system: Robotic-assisted gallbladder removal (De Novo) • Perimeter B-Series OCT with ImgAssist AI 2.0: Real-time surgical margin assessment during breast cancer surgery (PMA under review) • Technology with steep learning curve	Pre-market Approval (PMA) or De Novo • Extensive clinical trials • Comprehensive safety analysis • Risk mitigation protocols • Mandatory training programs	Pre-market: • Multi-phase clinical trials (n ≥ X # of procedures) • Safety and efficacy outcomes • Failure/error mode analysis • Extensive cybersecurity assessment • Long-term outcomes data Clinical Evidence: • Superiority or non-inferiority • Comprehensive safety profile • Risk–benefit analysis	Reporting: • Adverse event reporting (must be within X hours of critical incident) • Monthly performance reports • Comprehensive outcome tracking Performance: • Frequent, periodic real-world evidence • Mandatory registry participation • Automatic shutdown triggers
Key regulatory principles	Performance standards: • Standardized metrics for each technology type • Technical and clinical performance requirements • Real-world validation across diverse populations • Continuous monitoring with intervention thresholds		Safety Requirements: • Procedural risk assessment (irreversible actions) • Failure/error detection and automatic fallback systems • Comprehensive cybersecurity protection • Mandatory user training and competency verification	

## Three tier risk classification system

### Low risk technologies

Low risk technologies include digital surgery technologies that pose minimal potential for direct patient harm (Table 1). This includes training simulators, basic surgical planning software without real-time guidance, and data analytics platforms for quality improvement. Examples include AI-enabled imaging analysis tools such as Aidoc's BriefCase, which screens CT scans for incidental findings, and Viz Vascular Suite, which detects vascular pathologies to aid in workflow prioritization [56]. These technologies should undergo streamlined regulatory pathways that have reduced documentation requirements but still should include mandatory post-market surveillance. The regulatory burden should focus on basic safety validation, cybersecurity standards, and user interface design factors.

### Moderate risk technologies

Moderate risk technologies include real-time surgical guidance systems, advanced visualization tools with decision support capabilities, and semi-autonomous instruments that require human oversight (Table 1). Examples include Proprio's Paradigm platform, which provides AI-guided visualization and real-time intraoperative measurements for spine surgery, and Moon Surgical's Maestro platform with ScoPilot, which enables AI-powered instrument tracking and laparoscope control during minimally invasive procedures [49, 50]. These technologies should require more comprehensive pre-market evaluation, including clinical validation studies, human factors assessment, analysis of learning curves, and algorithmic bias evaluation. There is a need for periodic but consistent performance monitoring with predefined intervention thresholds, and many of these technologies should include PCCPs. There should also be the requirement of evidence of safe integration within existing surgical workflows.

### High risk technologies

High risk technologies include autonomous or semi-autonomous surgical systems, AI-guided critical anatomical navigation, and any technology that can independently initiate irreversible actions (Table 1). Examples include novel robotic systems like the MIRA Surgical System, which represents the first miniaturized robotic-assisted surgery device cleared through De Novo for colectomy procedures, and CMR Surgical's Versius system for robotic-assisted gallbladder removal [51, 52]. The highest-risk category includes systems like Perimeter's B-Series OCT with ImgAssist AI 2.0, which provides real-time assessment of surgical margins during breast cancer surgery and is currently under review via the PMA pathway due to its novel application and direct impact on immediate surgical decision-making [53]. These require the most stringent level of regulation through either De Novo or PMA pathways with comprehensive safety and risk mitigation protocols in addition to rigorous randomized clinical trials. For these trials, there should also be hybrid effectiveness-implementation designs as AI interventions’ effectiveness is often context-specific, making implementation measures one in the same with effectiveness measures [29].

### Predetermined change control plans

A critical component of AI regulation that is relevant regardless of risk levels, are PCCPs. PCCPs represent a proactive regulatory approach that addresses the adaptive nature of AI-enabled devices by establishing pre-authorized pathways for modifications that would otherwise require additional FDA submissions [15]. For companies developing digital surgery technologies that incorporate continuous learning algorithms or anticipate iterative improvements, PCCPs provide regulatory efficiency without compromising patient safety oversight.

These plans must specify the Description of Modifications that can be made, establish a detailed Modification Protocol with predefined acceptance criteria, and include a comprehensive Impact Assessment that evaluates benefits and risks [15]. Particularly for moderate and high-risk digital surgery technologies, PCCPs enable manufacturers to implement algorithm refinements, performance optimizations, and safety enhancements in response to real-world data while maintaining continuous regulatory compliance. Without well-designed PCCPs, the iterative nature of AI-enabled surgical technologies could create significant regulatory bottlenecks that delay beneficial improvements from reaching patients, particularly as these systems learn and adapt from clinical use and new data points.

## Performance metrics and measurement standards

A major barrier to effective digital surgery regulation is the lack of standardized, clinically meaningful performance metrics. Evaluation approaches that overemphasize algorithmic outputs and prioritize inappropriately defined technical metrics like sensitivity and specificity without linking them to clinical or patient-centered outcomes risk approving systems that perform well in silico but fail to improve care delivery or safety in practice [57, 58]. In surgery, this disconnect is particularly concerning, as technical metrics may inadequately capture workflow integration, error prevention, or ergonomic benefits that ultimately influence patient outcomes [59]. Furthermore, performance metrics frequently fail to reflect what matters most in the specific clinical application, inadequately measuring meaningful scientific progress and limiting practical translation of AI techniques [16, 41, 59]. Regulatory frameworks must balance evaluation criteria that assess both technical performance, quality, and clinical impact.

Technical performance metrics should be tailored to the modality and intended use of AI systems. For example, registration precision is critical for AR navigation platforms, latency thresholds define usability in real-time guidance systems, and fail-safe detection is paramount for autonomous robotic functions [60]. Importantly, validation must extend beyond single-institution datasets to include diverse patient populations and clinical environments to ensure generalizability and mitigating risks of algorithmic bias [61, 62]. Surgeon trust and usability should also be incorporated as performance indicators, given that clinician adoption determines real-world effectiveness.

Clinical performance standards should demonstrate meaningful improvement in surgical outcomes, quality, efficiency, technical performance, and patient safety. This requires moving beyond traditional randomized controlled trials to include pragmatic trial designs that reflect real-world workflow constraints, adaptive study protocols to accommodate evolving algorithms, and long-term tracking of outcomes, such as complications, readmissions, and costs that extend beyond immediate procedural success [29, 63]. For example, in laparoscopic cholecystectomy, AI tools designed to highlight the critical view of safety (CVS) should not only show high recognition accuracy but also demonstrate reductions in bile duct injury rates and operative errors across multiple diverse sites [64].

Because AI systems may experience performance drift as surgical techniques evolve or new patient populations are encountered, post-market surveillance for adverse events and user satisfaction is essential [25]. Standards should include automated reporting mechanisms, error logging, standardized incident classification systems, and clear escalation procedures when performance thresholds are not met [60].

### Evaluation frameworks for digital surgery

While digital surgery tools present unique considerations and require tailored pathways, we have established frameworks, guidelines, and principles to build on. The IDEAL framework provides a critical foundation that these technologies should progress through at each development stage [30, 31]. Although this framework does not address some unique challenges of AI-based systems, groups can work together to tailor the framework, as demonstrated by the IDEAL Robotics Colloquium's specific recommendations for surgical robotics development [32].

The IDEAL framework encompasses five stages: stage 1 (idea), stage 2a (development), stage 2b (exploration), stage 3 (assessment), and stage 4 (long-term monitoring). For AI-integrated robots, Marcus and colleagues recommend initially examining AI components separately, followed by in silico and simulator-based assessment (IDEAL stage 0), then first-in-human studies and clinical evaluation using reporting guidelines like DECIDE-AI (Developmental and Exploratory Clinical Investigations of DEcision support systems driven by Artificial Intelligence) (stages 1—2a) [32, 33]. DECIDE-AI is a stage-specific reporting guideline for early and live clinical evaluation of decision support systems [65]. Next, collaborative prospective cohort studies across various settings (stage 2b) should establish expert consensus on the nature of the patients and procedures to be studied in trials and what performance metrics are most important. Then the version can more to randomized comparative studies against an appropriate control group (stage 3). Following widespread adoption, long-term real-world performance monitoring becomes the focus (stage 4).

Beyond the IDEAL framework for surgical innovation, other relevant frameworks can be utilized depending on the technology's development stage [33]. For preclinical development, TRIPOD-AI (Transparent Reporting of a multivariable prediction model for Individual Prognosis Or Diagnosis) can guide reporting of prediction model development, validation, and updates [66]. STARD-AI (Standards for Reporting Diagnostic Accuracy) provides guidance for reporting diagnostic accuracy studies [67]. For comparative prospective evaluation, SPIRIT-AI (Standard Protocol Items: Recommendations for Interventional Trials) can be used to guide reporting protocols of randomized controlled trials evaluating AI systems as interventions [68, 69], while CONSORT-AI (Consolidated Standards of Reporting Trials) focuses on reporting effectiveness and safety of randomized controlled trials for AI systems [69, 70].

### Role of professional societies and collaborative communities

While manufacturers generate technical evidence for regulatory submissions, professional societies are uniquely positioned to ensure evaluation metrics reflect clinically meaningful endpoints. For clinical performance standards and safety monitoring requirements, professional societies such as the American College of Surgeons (ACS), the Society of American Gastrointestinal and Endoscopic Surgeons (SAGES), and the European Association for Endoscopic Surgery (EAES) have the expertise to define consensus standards for surgical AI and ensure evaluation metrics reflect real-world surgical priorities and patient safety concerns.

These societies could go a step further to form collaborative communities. Collaborative communities convene diverse stakeholders to co-develop best practices, clarify poorly defined challenges, generate and evaluate evidence for new approaches, and establish shared benchmarks [71]. Formally recognized by the FDA, these communities are forums where public and private sector members work together to achieve common objectives and solve shared challenges related to medical device development and regulation [71]. They also may work to clarify ill-defined challenges or generate consensus on the definition and scope of the challenge which can aid in tailoring appropriate strategies to tackle those challenges [71]. These communities would best be convened by interested stakeholders. For digital surgery regulation, these stakeholders could include surgeons, device manufacturers, patients, healthcare system leaders, and FDA representatives to collectively determine and recommend appropriate performance metrics and evaluation standards.

The value of these professional societies and collaborative approaches lies in their ability to identify what metrics matter most in real clinical scenarios. For example, consider an AI-enabled device that detects melanoma [72]. A device with 90% sensitivity and specificity might initially sound inferior to one with 96% sensitivity but only 30% specificity. However, clinical expertise becomes critical here because the device with the higher sensitivity is actually preferable in this scenario due to the fact that missing a melanoma diagnosis can have devastating consequences, including death. When melanoma is caught while localized, the 5-year survival rate exceeds 99%, but if the cancer spreads to distant parts of the body, the 5-year survival rate plummets to only 35% [73]. Conversely, for detecting other skin lesions where the consequences of missing the diagnosis are much less severe, specificity becomes more important because clinicians want to balance unnecessary biopsies or treatments with identification.

This example demonstrates how clinical perspective shapes which performance metrics matter most for patient safety and outcomes. In minimally invasive surgery (MIS), examples that determine which outcomes are most critical for AI validation may include reliable recognition of the critical view of safety during cholecystectomy, identification of high-risk anatomical landmarks to prevent intraoperative injury, and workflow efficiency improvements that reduce cognitive burden [64, 74]. These examples balance optimal metrics based on disease severity and treatment implications.

While manufacturers may be able to demonstrate impressive technical performance metrics for surgical technologies, surgical societies and collaborative communities can ensure the evaluation criteria capture the most critical clinical aspects of procedures. By aligning technical rigor with clinical priorities, these societies and communities can accelerate safe adoption of digital tools while ensuring that regulatory decisions are grounded in patient outcomes and surgical safety.

### Evidence requirements and burden of proof

The burden of proof for digital surgery technologies must reflect the irreversible nature of surgical procedures and the potential for significant, immediate, and permanent patient harm. This necessitates comprehensive evidence generation before market entry and continued validation throughout the product lifecycle.

Pre-market evidence requirements should continue to be tiered based on risk classification. Low risk technologies can require basic safety validation and usability testing, while high-risk systems necessitate extensive clinical trials with statistically powered safety endpoints. The European CORE–MD consortium (Coordinating Research and Evidence for Medical Devices) has developed a practical clinical risk score that provides a structured approach to determining appropriate evidence requirements for AI-enabled medical devices, which we could build on [40].

This scoring system evaluates three key domains. First is a valid clinical association, which considers how transparency and oversight can be achieved, and that there is a clear and valid association of the technology with its targeted indication. Second is valid technical performance, which considers the strength of validation and how rigorously the technology has been tested. Third is clinical performance, which considers the use context (i.e., disease type, condition, healthcare situation) and the function of the output (i.e., inform vs drive vs diagnose or treat).

Higher composite scores indicate devices that require extensive clinical investigations before regulatory approval, while lower scores suggest devices could be approved with less rigorous pre-market evaluation. This scoring system could be adapted for digital surgery technologies in the United States to ensure that regulatory requirements are proportionate to actual risk. Regardless of the risk tier, the evidence should demonstrate not only that the technology performs as intended in controlled settings, but that it integrates safely with existing surgical workflows, does not introduce increased cognitive burdens for surgeons, and ultimately improves patient outcomes rather than simply performs well with technical metrics.

Post-market evidence generation and additional data collection should also be mandatory for all these technologies [40], with reporting requirements that scale with risk level. Low risk technologies should report basic usage patterns and adverse events, while high-risk systems should require comprehensive real-world evidence collection including detailed outcome tracking, performance monitoring, and comparative effectiveness studies. Digital surgery technologies must prove their effectiveness not just in controlled research environments, but they must prove effective in the context of their intended use [75]. This requires evidence collection across multiple institutions, diverse patient populations, and varying levels of user expertise.

## Conclusions

Digital surgery is rapidly transforming surgical care, but its adaptivity and autonomy demand new regulatory approaches. Regulation of digital surgery must balance innovation with safety, recognizing that both excessive and insufficient regulation can harm patients. Traditional metrics and trial designs are insufficient, as evaluation must be anchored in clinically meaningful outcomes, ongoing surveillance, and real-world validation. Professional societies and collaborative communities are essential in defining these standards, ensuring that innovation aligns with surgical priorities and patient safety. The risk-stratified approach outlined here provides a foundation for achieving this balance through evidence-based standards, appropriate burden of proof requirements, and evaluation processes that address the unique challenges of digital surgery technologies.
